# Correlation between Cardiopulmonary Indices and Running Performance in a 14.5 km Endurance Running Event

**DOI:** 10.3390/ijerph191912289

**Published:** 2022-09-27

**Authors:** Milena Tomovic, Alexandros Toliopoulos, Nikolaos Koutlianos, Anastasios Dalkiranis, Sasa Bubanj, Asterios Deligiannis, Evangelia Kouidi

**Affiliations:** 1 Sports Medicine Laboratory, School of Physical Education and Sports Science, Aristotle University, Thermi PC, 57001 Thessaloniki, Greece; 2 Faculty of Sport and Physical Education, University of Nis, 18000 Nis, Serbia

**Keywords:** running, maximal oxygen uptake, running economy, sports performance, cardiopulmonary exercise test

## Abstract

Background: Running is a common recreational activity, and the number of long-distance-race participants is continuously growing. It is well-established that regular physical activity can prevent and manage non-communicable diseases and benefit public health. Training for a long-distance race requires development of specific aerobic abilities and should generate the desired race performance. The purpose of this study was to support the training design and motivation of recreational endurance runners, by investigating whether a 14.5 km race performance of long-distance runners correlates with their cardiopulmonary indices measured in the laboratory. Methods: To examine the relationships of a 14.5 km running performance with the cardiopulmonary parameters of amateur runners, a cross-sectional study design was applied. Fifteen (eleven men and four women) recreational long-distance runners (aged 41.3 ± 9.2 years) from Northern Greece were included in the study and were evaluated in the laboratory within one week before an endurance running race—the 14.5 km Philip Road race, in Greece. The laboratory-based examinations of the athletes consisted of a comprehensive medical pre-participation screening and maximal cardiopulmonary exercise testing. Results: The results showed that the 14.5 km race performance time (73.8 ± 9.7 min) significantly correlated with the cardiopulmonary-exercise-testing speed-related indices at specific submaximal and maximal workloads (*p* < 0.01, *p* < 0.05), while the cardiopulmonary indices of oxygen uptake did not reliably predict race running time (*p* > 0.05). Conclusions: There is a better correlation of the 14.5 km running performance of recreational long-distance runners with the cardiopulmonary-exercise-testing speed-related indices at specific workloads than with the indices of oxygen uptake, running economy or respiratory economy. When preparing a training strategy, amateur long-distance runners should mostly rely on specific running-speed-related laboratory data rather than on oxygen-uptake values.

## 1. Introduction

Running is a common recreational activity, and the number of long-distance-race participants is continuously growing [1,2,3,4,5]. In England alone, more than 3 million adults participate in recreational running each month, and the USA and Australia show similar trends [6,7,8]. According to the Physical Activity Council (the USA’s definitive source for sports, fitness and recreational activity participation), running is one of the top 10 recreational activities that inactive Americans would choose, if about to commence regular exercise [8]. Regular physical activity is one of the cornerstones of public health, as it is proven to help prevent and manage non-communicable diseases such as hypertension, obesity and several cancers. An active lifestyle respects the environment and induces behaviours that can preserve and improve environmental health [7,9]. Regular running has confirmed health benefits [10,11,12,13,14], and a recent systematic review and meta-analysis indicates that running reduces the risk of all-cause, cardiovascular and cancer mortality by 27%, 30% and 23%, respectively [9]. The same research identifies a literature gap in studies that would include sustained running participation, reproducible assessment of running habits and accurate evaluation of running performance.

Training for a long-distance race requires development of specific aerobic abilities and should generate the desired race performance. Amateur runners often use elite runners’ training methodologies, risking external overload, with consequential higher incidence of overuse injuries [15,16,17]. Furthermore, the studied relationship between recreational runners’ motivation and the incidence of injury emphasises the importance of adequate training methodology for injury prevention, especially among novice runners [17,18,19]. Thus, it is important to understand and objectively evaluate physiological and other parameters that, according to the existing literature, might influence or predict the long-distance running performance of amateur runners [20,21,22]. The available scientific evidence does not offer a reliable equation for performance prediction, and relevant studies are characterised with high data heterogeneity and often controversial findings. It is not clear if anthropometric [22], cardiorespiratory [21,23] or training load [21,24] indicators form valid performance-prediction models for popular long-distance amateur running events [20]. Additionally, performance prediction models do not identify the physiological parameters necessary for adequate training prescription. Data on anthropometric parameters are incomplete [22], while cardiopulmonary indices and training load indicators such as maximal oxygen uptake and kilometres of running per week, although studied repeatedly and with an immense amount of data, still do not provide the best solution for training design and follow up [22,23].

The purpose of this study was to support the training design and motivation of recreational endurance runners, by investigating whether a 14.5 km race performance of long-distance runners correlates with their cardiopulmonary indices measured in the laboratory. Hopefully, our findings will help recreational runners, not ready for or capable of a whole marathon race, reach the desired performance level.

## 2. Materials and Methods

To examine the relationships of a 14.5 km running performance with cardiopulmonary parameters of amateur runners, a cross-sectional study design was applied. Fifteen (eleven men and four women) recreational long-distance runners from Northern Greece were included in the study and were evaluated in the laboratory within one week before an endurance running race—the 14.5 km Philip Road race, Greece. The laboratory-based examinations of the athletes consisted of a comprehensive medical pre-participation screening and maximal cardiopulmonary exercise testing (CPET) performed on a treadmill. All amateur runners included were healthy and had been training regularly. They gave an informed consent and completed a questionnaire with detailed medical and training history. Participants’ training history will be presented in the results section.

Anthropometric parameters were measured prior to CPET (height—SECA Leicester resuscitation meter; weight, body fat and muscle mass percentage—Omron Karada Scan BF511, HBF-511T-E/HBF-511B-E). Maximal treadmill (Montara Trackmaster 428, KS, USA) CPET, via a breath-by-breath gas-analyzing system (Geratherm Respiratory GmbH’s BlueCherry, Bad Kissingen, Germany), followed the clinical exam in an appropriate laboratory environment (room temperature 20 °C and relative humidity between 25–50%). The gas-analyzing system was validated prior to each testing. Heart rate (HR) was monitored by Polar Receiver Pulstik system (Geratherm Respiratory GmbH, Bad Kissingen, Germany). Participants did not train nor consume any nutritional supplements or caffeinated beverages 24 h before testing. The maximal ramp exercise protocol [25] included: warm-up: 1 min at speed of 2 km/h, slope 0% and 3 min at speed of 5 km/h, slope 0%; test: speed 5 km/h and slope 1%. The workload was enhanced by a gradual increase in the speed, by 1.2 km/h per min, until exhaustion. Moreover, the slope increased to 2%, when participants reached a speed of 13 km/h, and did not increase further with speed increment beyond 13 km/h. Recovery was 5 min.

During the CPET, minute ventilation (VE, L/min), oxygen consumption (VO_2_, relative (mL/kg/min) and absolute values (mL/min)), respiratory exchange ratio (RER), VCO_2_ (carbon dioxide production, mL/kg/min), ventilatory equivalents for oxygen and carbon dioxide (VE/VO_2_, VE/VCO_2_), PETO_2_ (end tidal oxygen volume), PETCO_2_ (end tidal carbon dioxide volume), HR, oxygen pulse (O_2_ pulse, mL/beat), time to voluntary exhaustion (4 min of warm up and 5 min of recovery were excluded) and the 2nd ventilatory threshold (VT) were measured [26]. Additionally, the same parameters as well as the running speeds (“v” expressed in km/h) were noted at the VT and RER1 (RER value of 1, VO_2_ = VCO_2_) points of the CPET: VO_2VT_, VO_2RER1_, vVT, vRER1, HR_VT_, HR_RER1_ and maximal parameters: VO_2_max, vVO2max, vpeak, HRmax, VEmax, max O_2_ pulse, RERmax, tidal volume max, VO_2_max/VO_2_ref% (maximal VO_2_ expressed as a percentage of a VO_2_max value predicted according to participant’s age, sex and training habits) and VT/VO_2_max% (percentage of achieved maximal VO_2_ at VT CPET’s point).

Running economy (RE)8, RE10, RE12, VO_2_/WR and VO_2_/WR (7.9–13.1 km/h) values were used as running economy indicators. The RE8, RE10 and RE12 indicators were derived from VO_2_ of each athlete at speeds of 8 km/h, 10 km/h and 12 km/h, respectively, and were expressed in mL/min/kg. The VO_2_/WR and VO_2_/WR (7.9–13.1) indicators were calculated directly from the ergospirometer, and they represent VO_2_ achieved at these speeds and converted to a work rate (WR) expressed in watts (mL/min/watt). The VO_2_/WR index represents the average value through the whole test load, while the VO_2_/WR (7.9–13.1) index represents the average oxygen uptake per work rate between speeds of 7.9 km/h and 13.1 km/h. These specific values of speeds were marked because they were reached by all participants; thus, a direct comparison between them was possible. Furthermore, the speed of 7.9 km/h is the lowest intensity that forced running over walking.

Two weeks after the lab evaluation, the athletes participated in the Philip Road race—14.5 km route from Vergina to Veria, in Greece. The competition was entirely on asphalt, and it started at 10.30 am. The athletes were offered water at 5.4 km and water and isotonic fluid at 10.4 km. The weather on the race day was clear and with optimal conditions. The race performance times of the runners were collected from the official results, and these time records were net, i.e., the time from the moment the athletes crossed the starting line to the time they arrived at the finish line.

Descriptive statistics were used to describe categorical variables. Continuous variables were expressed as mean ± SD and Shapiro–Wilk test was used for testing the normality of all data. The differences between values were evaluated using the paired sample t-test. Relationships between categorical variables were tested using the chi-squared statistic. The Pearson linear correlation coefficient was used for quantitative values, and, for the non-linear data, we used Spearman non-parametric correlation. Statistical analysis was carried out with the IBM SPSS statistical program (Social Package for Social Sciences, Chicago, IL, USA, version 25.0). A two-tailed *p* < 0.05 was accepted as statistically significant.

## 3. Results

All fifteen recruited recreational runners (aged 41.3 ± 9.2 years) completed the 14.5 km race and had no injuries or any health disorders during the race. They had been practicing running for the past 5.6 (±5.6) years, with a frequency of 5 (±1.4) days, 7.2 (±3.1) hours and 52.7 (±19.5) km per week. Their demographic, anthropometric and race performance data, as shown in Table 1 and Table 2, contain the overview of the participants’ CPET results.

Correlation analysis of the anthropometric parameters showed, for the race performance times, significant coefficients only for the height (174.5 ± 7.8 cm, r = −0.534, *p* < 0.01) and the muscle mass percentage (35.6 ± 3.2%, r = −0.696, *p* < 0.01) of the participants. From the participants’ training history data, only the number of kms that participants achieved during their weekly training showed a statistically significant negative correlation (52.7 ± 19.5 km, r = −0.640, *p* < 0.05) with the race performance time.

**Figure 1:**
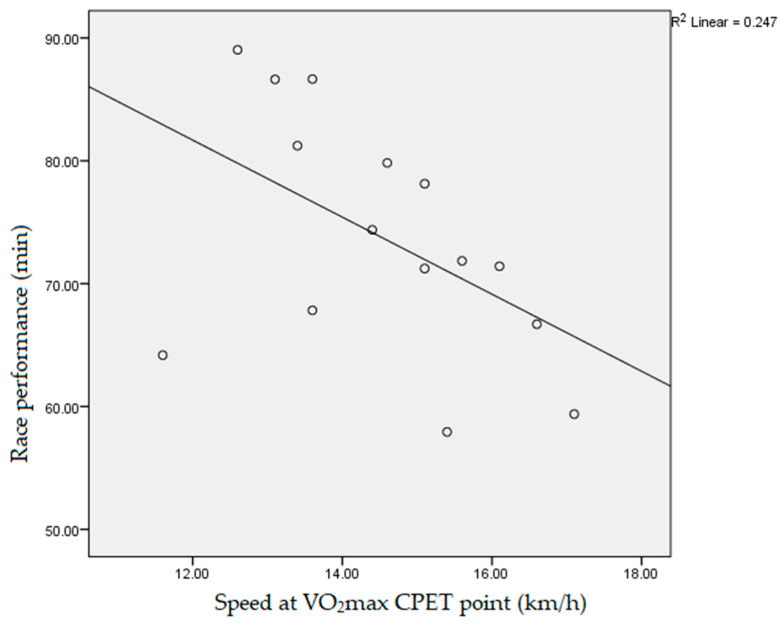
Correlation between participants’ speed at maximal oxygen consumption point during cardiopulmonary exercise testing and race performance time (r = −0.531, *p* < 0.05). VO_2_max: maximal oxygen consumption; CPET: cardiopulmonary exercise testing.

**Figure 2:**
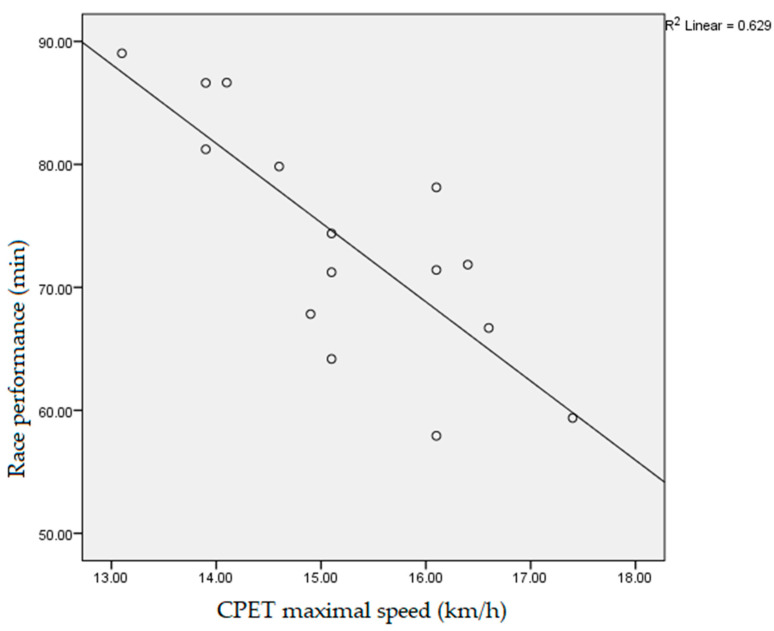
Correlation between participants’ maximal speed during cardiopulmonary exercise testing and race performance time (r = −0.754, *p* < 0.01). CPET: cardiopulmonary exercise testing.

**Figure 3:**
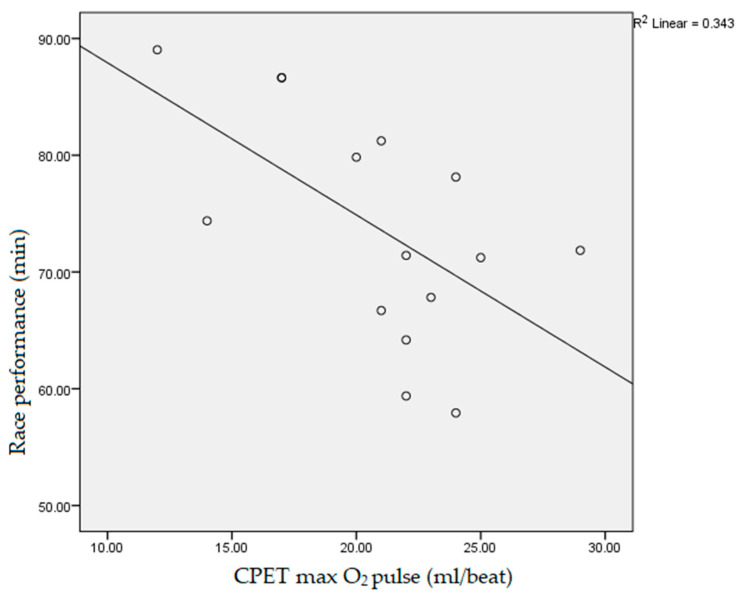
Correlation between participants’ maximal oxygen pulse during cardiopulmonary exercise testing and race performance time (r = −0.607, *p* < 0.05). CPET: cardiopulmonary exercise testing; max O_2_ pulse: maximal oxygen pulse.

**Figure 4:**
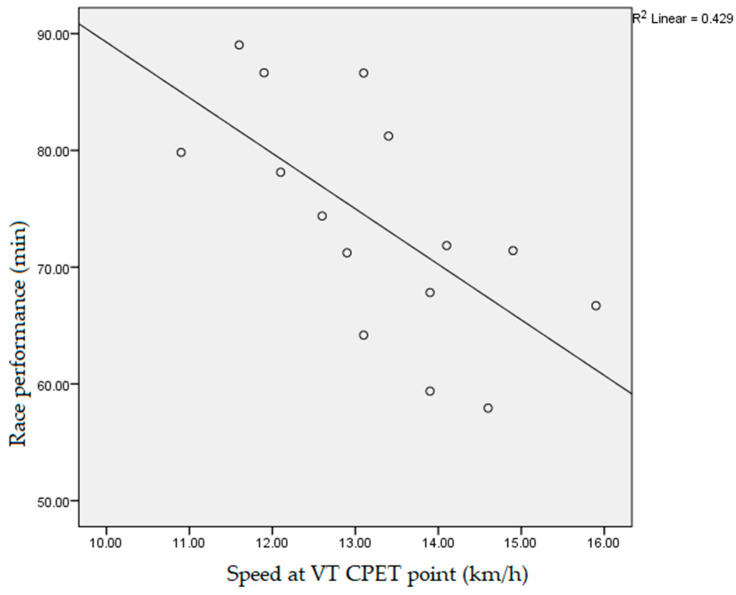
Correlation between participants’ speed at ventilatory threshold during cardiopulmonary exercise testing and race performance time (r = −0.733, *p* < 0.01). VT: ventilatory threshold; CPET: cardiopulmonary exercise testing.

**Figure 5:**
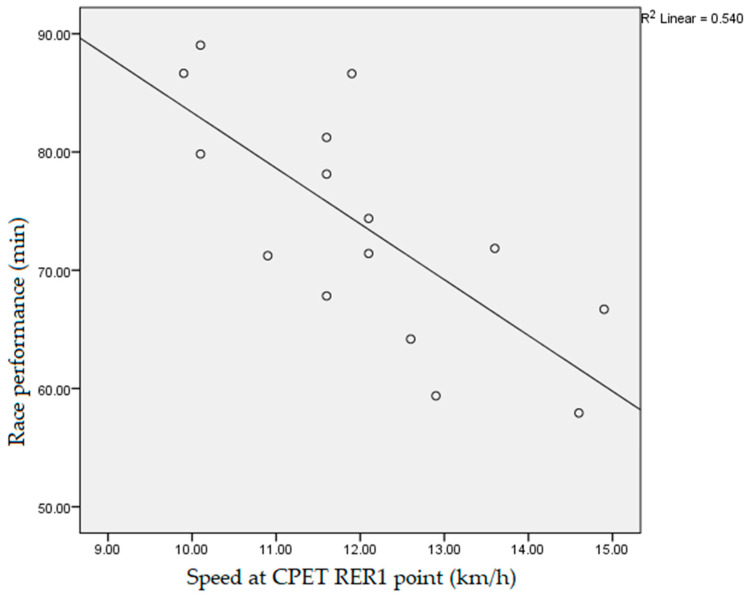
Correlation between participants’ speed at respiratory exchange ratio value (RER = 1) during cardiopulmonary exercise testing and race performance time (r = −0.671, *p* < 0.01). CPET: cardiopulmonary exercise testing; RER1: respiratory exchange ratio equal to one (RER = 1).

Data obtained during the participants’ CPET, which had a statistically significant negative correlation with the athletes’ race performance, measured as race end time, were: fatigue time (10.3 ± 1.2 min, r = −0.718, *p* < 0.01), vVO_2_max (14.5 ± 1.5 km/h, r = −0.531, *p* < 0.05, Figure 1), vpeak (15.2 ± 1.2 km/h, r = −0.754, *p* < 0.01, Figure 2), absolute VO_2_max (3.4 ± 0.5 L/min, r = −0.617, *p* < 0.05), max O_2_ pulse (20.9 ± 4.4 mL/beat, r = −0.607, *p* < 0.05, Figure 3) and tidal volume max (2.4 ± 0.4, r = −0.550, *p* < 0.05). The speed values (vVT, vRER) achieved by runners at the VT and RER1 points of their CPET had a significant (*p* < 0.01) negative correlation (r = −0.733, r = −0.671, respectively, Figure 4 and Figure 5) with the runners’ race performance times. Relative VO_2_max (46.2 ± 4.9 mL/min/kg, r = −0.422, *p* > 0.05), VO_2VT_ (42.5 ± 4.9 mL/min/kg, r = −0.390, *p* > 0.05), VT/VO_2_max% (92.1 ± 7.1%, r = −0.468, *p* > 0.05), VE (121.4 ± 17.4, r = −0.468, *p* > 0.05), VO_2_max/VO_2_ref% (130.2 ± 17.9%, r = 0.252, *p* > 0.0.5) and HR_VT_ (162.3 ± 14.9/min, r = 0.354, *p* > 0.05) did not show any statistically significant positive or negative correlation.

Running economy indicators were not significantly correlated with the race performance times of the studied runners: RE8 (27.2 ± 2.1 mL/min/kg, r = −0.036, *p* > 0.05), RE10 (33.2 ± 2.3 mL//min/kg), r = 0.232, *p* > 0.05), RE12 (38.5 ± 3.3 mL/min/kg), r = 0.079, *p* > 0.05), VO_2_/WR (7.1 ± 1.6 mL/min/watt, r = 0.164, *p* > 0.05) and VO_2_/WR (7.9–13.1) (5.2 ± 6.7 mL/min/watt, r = −0.181, *p* > 0.05).

## 4. Discussion

The results showed that the 14.5 km race performance time of recreational runners was significantly correlated with the running speeds achieved during CPET at VT, RER1, VO_2_max and the test peak point. The good predictive potential of specific running speeds (peak velocity, vVO_2_max, vVT) achieved during the exercise testing of recreational endurance runners was previously documented by a recent review [22] and other studies [21,27,28,29]. Running speeds associated with anaerobic threshold, maximal oxygen uptake and CPET peak values include, as determinants, both the aerobic and anaerobic attributes of the selected runners [22,27,30]. This can explain the great predictive potential of these parameters for a long-distance running event, which, in fact, requires both well-developed aerobic and anaerobic power [30]. Additionally, as already confirmed in the literature, the speed at the anaerobic threshold depends on both the maximal oxygen uptake and running economy [29,30,31]. Thus, the significant correlation of this specific speed with running performance, as noted in our study, is fully and favourably justified for separating the evaluation of the maximal oxygen uptake and running economy [29,30,31]. We can reasonably assume that the cumulative effect of the previous parameters shows a better predictive potential and responds to the complexity of long-distance-running’s physiological requirements.

The CPET indices of oxygen uptake (relative VO_2_max, VO_2VT_, VT/VO_2_max%, VO_2_max/VO_2_ref%) were not valid for predicting the performance times in our amateur long-distance runners. Only the absolute value of VO_2_max showed a significant correlation with race time. This finding should be carefully interpreted, since the VO_2_, in L/min, as a measure of aerobic capacity, highly depends on individual anthropometric characteristics [26]. Thus, the use of this value should not be considered as a primary index to predict the performance times in amateur long-distance runners.

The scientific literature has acknowledged the importance of oxygen uptake and aerobic power for endurance running activities, but amateur running performance prediction models remain dependent on running-speed indices and, according to some studies, anaerobic threshold and RE values [31,32,33,34]. Our study did not prove the utility of the calculated RE indicators, and studies with similar results concluded that RE is not useful in defining mid-distance running performance [28,35]. Furthermore, a recent systematic review and meta-analysis found that the existing evidence is not clear on the relationship of RE and mid- and long-distance running performance, as studies on this relationship have a high level of endogenous selection bias and an absence of allocation methodologies. Additional research is needed to evaluate the relationship between RE and running performance [36,37,38].

The participants’ CPET data for max O_2_ pulse demonstrated an interesting relationship with the race performance time. This parameter is traditionally used as a predictor of good cardiovascular health and for the evaluation and prognosis of ischemic cardiomyopathies. However, the existing scientific literature did not use max O_2_ pulse for performance prediction until now. O_2_ pulse depends on HR and oxygen uptake, and its maximal value is mainly determined by aerobic capacity—HRmax is age-related and does not depend on other individual physiological and pathological characteristics [26]. Future evaluation of max O_2_ pulse might be interesting for performance and health-related prognosis, especially in recreational athletic and patient populations. When the cardiovascular and respiratory fitness of physically active subjects is evaluated, it can be helpful from motivational and financial perspectives to produce, besides health indices, data that can predict or proclaim individual performance determinants [39,40]. Furthermore, physical fitness as a health determinant can help in defining tools for public health improvement, as it has been proven that regular physical activity can prevent and manage non-communicable diseases [7,11].

The available scientific evidence is heterogeneous regarding the utility of the anthropometric parameters of amateur long-distance runners for performance-prediction models [21,22]. Our study results on runners’ anthropometry should be evaluated in regard to that heterogeneity. Recreational runners differ in body composition, and their training and race participation does not require professional runners’ discipline and consequential body image. Thus, the study of anthropometric indices in amateur runners represents a great challenge for future research, since its utility may not be limited to only performance-prediction models. Anthropometric features of amateur mid- and long-distance runners can help define healthy and injury-free running guidelines. 

The outcomes of this study add valuable insight on amateur long-distance running performance and answer the literature’s need for a better understanding of the relationship between recreational runners’ cardiopulmonary indices and their training design. Our research highlights the cumulative effect of maximal oxygen uptake and running economy, expressed through running speeds, as better tools of training design for long-distance amateur running events. We define the cardiopulmonary indices suitable for the laboratory control of the training adaptations that are important for safe, motivated and injury-free recreational long-distance running. When designing the training strategy of amateur long-distance runners, one should use the running speeds achieved during CPET at VT, RER1, VO_2_max and the test peak point for progress control and periodization plan. The main limitations of the study were the small sample size and the absence of gender segregation. 

## 5. Conclusions

There is a better correlation of the 14.5 km running performance of recreational long-distance runners with CPET speed-related indices at specific workloads than with the indices of oxygen uptake, running economy or respiratory economy. When preparing a training strategy, amateur long-distance runners should mostly rely on specific running-speed-related laboratory data rather than on oxygen-uptake values.

## Figures and Tables

**Table 1:** Demographic, anthropometric and race performance characteristics of participants.

Participants	Race Performance (min)	Average Race Speed (km/h)	Age (Years)	Height (cm)	Weight (kg)	BMI ^b^ (kg/m^2^)	Body Fat (%)	Muscle Mass (%)
1	57.93	15.02	37	189	83	23.24	19	38.3
2	59.38	14.65	37	180.5	76	23.33	21.6	37
3	64.18	13.56	48	181	77	23.50	18.7	37.7
4	66.7	13.04	51	169	68	23.81	18.1	38.4
5	67.83	12.83	52	178	74	23.36	18.6	37.4
6	71.23	12.21	38	171	73	24.96	23	37.1
7	71.42	12.18	34	176	72	23.24	17.2	40.7
8	71.85	12.11	39	184	92	27.17	28.1	33.4
9	74.38	11.70	32	156.5	57	23.27	32.9	28.4
10	78.13	11.14	39	173.5	78	25.91	26	35
11	79.83	10.90	39	169.5	78	27.15	25.7	35.8
12	81.23	10.71	66	173	79	26.40	20.7	35.4
13	86.63	10.04	33	177	66	21.70	20.3	35.5
14	86.65	10.04	40	168.5	68	23.95	30.8	30.5
15	89.03	9.77	34	171	60	20.52	22.6	33.6
Mean Value	73.8	12.0	41.3	174.5	73.4	24.1	22.9	35.6
SD ^a^	9.7	1.6	9.2	7.8	8.8	2.0	4.8	3.2
Median	71.85	12.11	39	173.5	74.0	23.50	21.6	35.8

^a^ standard deviation; ^b^ body mass index.

**Table 2:** Cardiopulmonary exercise testing results of participants.

Participants	Test Time (min)	VE ^b^ (L/min)	Running Speed at VT ^c^ Point (km/h)	Maximal Running Speed	VO_2_max ^d^ (L)	VO_2_max ^d^ (mL/min/kg)	Oxygen Pulse (mL/Beat)	Maximal Heart Rate (Beat/min)
1	11.17	123	15.4	16.1	4.24	51.1	24	170
2	12.28	144	17.1	17.4	3.96	52.1	22	177
3	10.08	103	11.6	15.1	3.21	41.7	22	160
4	11.75	132	16.6	16.6	3.8	55.9	21	181
5	9.97	146	13.6	14.9	3.3	44.6	23	141
6	10.15	118	15.1	15.1	3.21	44.0	25	169
7	11.17	123	16.1	16.1	3.76	52.2	22	175
8	11.32	135	15.6	16.5	4.08	44.3	29	163
9	10.10	95	14.4	15.1	2.66	46.7	14	185
10	11.25	143	15.1	16.1	3.45	44.2	24	171
11	9.63	131	14.6	14.6	3.48	44.6	20	176
12	8.97	116	13.4	13.9	3.46	43.8	21	171
13	8.82	108	13.1	13.9	3.14	47.6	17	182
14	9.10	113	13.6	14.1	3.01	44.3	17	178
15	8.03	91	12.6	13.1	2.18	36.3	12	179
Mean Value	10.3	121.4	14.5	15.2	3.4	46.2	20.9	171.9
SD ^a^	1.2	17.4	1.5	1.2	0.5	4.9	4.4	11.0
Median	10.10	123.00	14.60	15.10	3.45	44.59	22.00	175.00

^a^ standard deviation; ^b^ minute ventilation; ^c^ ventilatory threshold; ^d^ maximal oxygen consumption.

## Data Availability

The data presented in this study are available on request from the corresponding author. The data are not publicly available due to the privacy of the included subjects.

## References

[1] Hulteen R.M., Smith J.J., Morgan P.J., Barnett L.M., Hallal P.C., Colyvas K., Lubans D.R. (2017). Global participation in sport and leisure-time physical activities: A systematic review and meta-analysis. Prev. Med..

[2] Borgers J., Breedveld K., Tiessen-Raaphorst A., Thibaut E., Vandermeerschen H., Vos S., Scheerder J. (2016). A study on the frequency of participation and time spent on sport in different organisational settings. Eur. Sport Manag. Q..

[3] Anthony D., Rüst C.A., Cribari M., Rosemann T., Lepers R., Knechtle B. (2014). Differences in participation and performance trends in age group half and full marathoners. Chin. J. Physiol..

[4] Knechtle B., Nikolaidis P.T., Zingg M.A., Rosemann T., Rüst C.A. (2016). Half-marathoners are younger and slower than marathoners. SpringerPlus.

[5] Lima M.G., Malta D.C., Monteiro C.N., da Silva Sousa N.F., Stopa S.R., Medina L.D.P.B., de Azevedo Barros M.B. (2019). Leisure-time physical activity and sports in the Brazilian population: A social disparity analysis. PLoS ONE.

[6] Stamatakis E., Chaudhury M. (2008). Temporal trends in adults’ sports participation patterns in England between 1997 and 2006: The health survey for England. Br. J. Sports Med..

[7] Eime R.M., Harvey J.T., Charity M.J., Casey M.M., Van Uffelen J.G.Z., Payne W.R. (2015). The contribution of sport participation to overall health enhancing physical activity levels in Australia: A population-based study. BMC Public Health.

[8] Wisconsin Office of Outdoor Recreation 2018 Participation Report: The Physical Activity Council’s Annual Study Tracking Sports, Fitness and Recreation Participation in the US. https://outdoorrecreation.wi.gov/Documents/Research%20Library%20Page%20files/US%20-%20Demographics%20%26%20Participation/Physical%20Activity%20Coucil%20Participation%20Report_2018.pdf.

[9] Pedisic Z., Shrestha N., Kovalchik S., Stamatakis E., Liangruenrom N., Grgic J., Titze S., Biddle S.J.H., Bauman A.E., Oja P. (2019). Is running associated with a lower risk of all-cause, cardiovascular and cancer mortality, and is the more the better? A systematic review and meta-analysis. Br. J. Sports Med..

[10] Lavie C.J., Lee D.C., Sui X., Arena R., O’Keefe J.H., Church T.S., Milani R.V., Blair S.N. (2015). Effects of running on chronic diseases and cardiovascular and all-cause mortality. Mayo Clin. Proc..

[11] Lee D.C., Brellenthin A.G., Thompson P.D., Sui X., Lee I.M., Lavie C.J. (2017). Running as a key lifestyle medicine for longevity. Prog. Cardiovasc. Dis..

[12] Wang Y., Lee D.C., Brellenthin A.G., Eijsvogels T.M., Sui X., Church T.S., Lavie C.J., Blair S.N. (2019). Leisure-time running reduces the risk of incident type 2 diabetes. Am. J. Med..

[13] Hespanhol J.L.C., Pillay J.D., van Mechelen W., Verhagen E. (2015). Meta-analyses of the effects of habitual running on indices of health in physically inactive adults. Sport Med..

[14] Lee D.C., Pate R.R., Lavie C.J., Sui X., Church T.S., Blair S.N. (2014). Leisure-time running reduces all-cause and cardiovascular mortality risk. J. Am. Coll. Cardiol..

[15] Videbæk S., Bueno A.M., Nielsen R.O., Rasmussen S. (2015). Incidence of running-related injuries per 1000 h of running in different types of runners: A systematic review and meta-analysis. Sport Med..

[16] Kluitenberg B., van Middelkoop M., Diercks R., van der Worp H. (2015). What are the differences in injury proportions between different populations of runners? A systematic review and meta-analysis. Sport Med..

[17] Gauffin H., Tillander B., Dahlström Ö., Lyth J., Raysmith B., Jacobsson J., Timpka T. (2019). Maintaining motivation and health among recreational runners: Panel study of factors associated with self-rated performance outcomes at competitions. J. Sci. Med. Sport.

[18] León-Guereño P., Tapia-Serrano M.A., Sánchez-Miguel P.A. (2020). The relationship of recreational runners’ motivation and resilience levels to the incidence of injury: A mediation model. PLoS ONE.

[19] Linton L., Valentin S. (2018). Running with injury: A study of UK novice and recreational runners and factors associated with running related injury. J. Sci. Med. Sport.

[20] Keogh A., Smyth B., Caulfield B., Lawlor A., Berndsen J., Doherty C. (2019). Prediction equations for marathon performance: A systematic review. Int. J. Sports Physiol. Perform..

[21] Gómez-Molina J., Ogueta-Alday A., Camara J., Stickley C., Rodríguez-Marroyo J.A., García-López J. (2017). Predictive variables of half-marathon performance for male runners. J. Sport Sci. Med..

[22] Boullosa D., Esteve-Lanao J., Casado A., Peyré-Tartaruga L.A., Gomes da Rosa R., Del Coso J. (2020). Factors affecting training and physical performance in recreational endurance runners. Sports.

[23] Esteve-Lanao J., Del Rosso S., Larumbe-Zabala E., Cardona C., Alcocer-Gamboa A., Boullosa D.A. (2021). Predicting recreational runners’ marathon performance time during their training preparation. J. Strength Cond. Res..

[24] Salinero J.J., Soriano M.L., Lara B., Gallo-Salazar C., Areces F., Ruiz-Vicente D., Abián-Vicén J., González-Millán C., Del Coso J. (2017). Predicting race time in male amateur marathon runners. J. Sports Med. Phys. Fit..

[25] Winter U.J., Gitt A.K., Fritsch J., Berge P.G., Pothoff G., Hilger H.H. (1994). Methodologic aspects of modern, computerized ergospirometry (CPX): Ramp program, constant workload test and CO_2_ rebreathing method. Z. Kardiol..

[26] Wasserman K., Hansen J.E., Sue D.Y., Stringer W.W., Whipp B.J. (2004). Principles of Exercise Testing and Interpretation: Including Pathophysiology and Clinical Applications.

[27] Scott B.K., Houmard J.A. (1994). Peak running velocity is highly related to distance running performance. Int. J. Sports Med..

[28] Bragada J.A., Santos P.J., Maia J.A., Colaço P.J., Lopes V.P., Barbosa T.M. (2010). Longitudinal study in 3,000 m male runners: Relationship between performance and selected physiological parameters. J. Sport Sci. Med..

[29] Nicholson R.M., Sleivert G.G. (2001). Indices of lactate threshold and their relationship with 10-km running velocity. Med. Sci. Sports Exerc..

[30] Billat L.V., Koralsztein J.P. (1996). Significance of the velocity at VO_2max_ and time to exhaustion at this velocity. Sport Med..

[31] Bassett D.R., Howley E.T. (2000). Limiting factors for maximum oxygen uptake and determinants of endurance performance. Med. Sci. Sports Exerc..

[32] Conley D.L., Krahenbuhl G.S. (1980). Running economy and distance running performance of highly trained athletes. Med. Sci. Sports Exerc..

[33] Daniels J., Daniels N. (1992). Running economy of elite male and elite female runners. Med. Sci. Sports Exerc..

[34] Bassett D.R., Howley E.T. (1997). Maximal oxygen uptake: “Classical” versus “contemporary” viewpoints. Med. Sci. Sports Exerc..

[35] Grant S., Craig I., Wilson J., Aitchison T. (1997). The relationship between 3 km running performance and selected physiological variables. J. Sports Sci..

[36] Borgen N.T. (2018). Running performance, VO_2max_, and running economy: The widespread issue of endogenous selection bias. Sport Med..

[37] O Sullivan I.J., Johnson M.I., Hind K., Breen S., Francis P. (2019). Are changes in running economy associated with changes in performance in runners? A systematic review and meta-analysis. J. Sports Sci..

[38] Alvero-Cruz J.R., Carnero E.A., García M.A.G., Alacid F., Correas-Gómez L., Rosemann T., Nikolaidis P.T., Knechtle B. (2020). Predictive performance models in long-distance runners: A narrative review. Int. J. Environ. Res. Public Health.

[39] Lee L.L., Arthur A., Avis M. (2008). Using self-efficacy theory to develop interventions that help older people overcome psychological barriers to physical activity: A discussion paper. Int. J. Nurs. Stud..

[40] André N., Agbangla N.F. (2020). Are barriers the same whether I want to start or maintain exercise? A narrative review on healthy older adults. Int. J. Environ. Res. Public Health.

